# European Federation for Exploratory Medicines Development Lyon Conference 2019: The Changing Landscape of Early Medicines Development—Be Prepared

**DOI:** 10.3389/fphar.2019.01377

**Published:** 2019-11-29

**Authors:** Tim Hardman, Henri Caplain, Sylvie Rottey, Desiree Douglas, Steffan Stringer, Hildegard Sourgens

**Affiliations:** ^1^Association for Human Pharmacology in the Pharmaceutical Industry (AHPPI), Richmond, United Kingdom; ^2^Association Francaise de Pharmacologie Translationelle (AFPT), Paris, France; ^3^Belgian Association of Phase-1 Units (BAPU), Brussels, Belgium; ^4^Association for Applied Human Pharmacology (AGAH), Hamburg, Germany

**Keywords:** early phase clinical drug development, the European Federation for Exploratory Medicines Development, conference report, EUFEMED, pharmacokinetics

## Abstract

The second biennial conference of the European Federation for Exploratory Medicines Development (EUFEMED) was the result of a continued effort of EUFEMED to gather all stakeholders of exploratory clinical drug development to evaluate and discuss recent developments in the field. The conference focused on how the landscape around early clinical development is changing and how clinical pharmacologists might prepare for these changes. A preconference workshop gave consideration to the impact that modeling and simulation, including physiology-based pharmacokinetic strategies, is having on the practice of clinical development. A second workshop looked at the challenges introduced by biological agents. The keynote address explored the potential role of virtual trials in early medicines development with emphasis on how models can help to understand and inform the drug development process. Presentations that followed covered a broad range of subjects including the potential impact of digital support on study performance in early phase development, extending from recruitment to remote data collection, lay person summaries, data transparency, and ethical considerations for trials in healthy subjects. The second day of the conference focused on future regulatory challenges in the field of early clinical development (including Brexit) and how to prepare for changes in the landscape. Subjects covered included new approaches and designs in oncology, the introduction of more complex study designs and digital biomarkers. Presentations given by invited speakers are published at https://www.eufemed.eu/download-presentations-of-the-lyon-conference-2019/.

## Introduction

The European Federation for Exploratory Medicines Development (EUFEMED, www.eufemed.eu) is a not for profit federation that aims to improve and strengthen the early phase clinical drug development process in Europe. Its second biennial conference was held from 15^th^ to the 17^th^ May 2019 in Lyon (France). The 3-day meeting included a mixture of focused scientific sessions, interactive workshops, and open forum discussions reflecting recent developments in the changing landscape of early medicines development.

This report summaries the key learnings from an audience perspective derived from the conference.

### Preconference Workshops

#### Workshop 1: Modeling and Simulations, Including Physiology-Based Pharmacokinetic Strategies to Improve the Clinical Development

A growing number of regulatory submissions include physiologically based pharmacokinetic (PBPK) models that require the use of specialized software platforms. Although PBPK modeling is presently mentioned in several guidelines issued by the European Medicines Agency (EMA), there is limited understanding on what to include in a PBPK modeling report including, in particular, details of the predictive performance of the drug model (European Medicines Agency Science Medicines Health, 2018a). FrançoisBouzom (UCB BioPharm, Belgium) introduced the principles of modeling and simulation including PBPK. He reasoned that to succeed in treating a disease it is necessary to define the *right* drug with the *right* dose regimen administered to the *right* patient—the three R's. To build effective models it is necessary to combine the data and understanding obtained through a variety of tools and experiments. In his presentation, Dr Bouzom summarized the principles, values, and limitations of various modeling approaches currently being used to empower assumption testing through simulations at different stages of development. He concluded that although models can range from descriptive to fully mechanistic they must fit with their intended purpose.

In extending the concept of modeling, Roberto Gomeni (Pharmacometrica, France) discussed general *in silico* frameworks for maximizing the benefit-risk ratio of a treatment. The net benefit of a treatment is usually defined by the relationship between potential clinical improvement and the risk of adverse events (Gomeni et al., 2019). He introduced the concept of convolution-based modeling as a means of optimizing the potential clinical benefit of new pharmacological treatments. Dr Gomeni discussed how it is possible to optimize benefit-risk ratio by identifying the optimal dose and/or dose regimen along with its best performing *in vivo* release properties. A general *in silico* tool was presented that can be used to investigate the *in vitro* and *in vivo* release properties required to maximize the benefit-risk ratio—employing a convolution-based, exposure-response model that includes surface response analysis. The presentation concluded that model-informed approaches can provide a methodological framework for developing drugs with the optimal dose and delivery characteristics to provide clinical benefits.

Géraldine Ayral (Lixoft, France) reviewed simulation of first-in-human testing using an allometrically scaled, population mechanistic model. Whereas simple allometric scaling of clearance and volume is often sufficient for small candidate molecules, the nonlinear pharmacokinetic nature of many biologics necessitates the use of more advanced methods when predicting how they might behave in humans. Model-based approaches, integrating as much mechanistic information as possible, have proven to be of excellent predictive value. Dr Ayral gave an illustration of how such an analysis might be performed using cynomolgus monkey data for the human immunoglobulin G (IgG)2 monoclonal antibody, PF-03446962. The comparison of the predictions with real phase I data demonstrated how predictions can come close (within 1–2-fold) to clinical observations. She concluded that such modeling and simulation workflows are relatively straightforward and easy to implement.

Pauline Traynard (Lixoft, France) mapped the simulation and extension of population pharmacokinetic models obtained during phase I studies to establish phase II trial design. She reminded the audience that efficacy trials are expensive and time consuming, whereas using pharmacokinetic/pharmacodynamic models in combination with predictive tools can accelerate drug development by considering options *in silico* simulations. Using modeling and simulations on phase I pharmacokinetic data for a dopamine reuptake inhibitor, Dr Traynard demonstrated how it is possible to use them cost effectively to inform the design of phase II clinical trials. It was demonstrated how the proposed model first permits interpolation and assessment of relevant clinical endpoints, accounting for inter-individual variability and estimation uncertainty. Extrapolation beyond the conditions of the phase I trial allowed the identification of the optimal doses and trial designs that were likely to offer putative predictions of effect response and alternate routes of administration.

Lars Kuepfer (Bayer AG, Germany) reviewed how PBPK modeling combined with Bayesian statistics and targeted clinical data can predict drug pharmacokinetics across patient populations. He noted how translation of knowledge between different preclinical and clinical phases is a key challenge in pharmaceutical development programs; but that computational modeling may one day inform the development of systematic concepts for processing, curation, and re-evaluation of information and data. PBPK models integrate experimental data from different layers of biological organization to describe mechanistically the physiological processes underlying drug administration, distribution, metabolism, and excretion. Examples were provided on how model-based approaches for clinical translation can help to translate preclinical findings from animal models to healthy human volunteers and how to further bridge between different patient subgroups.

Various examples for the integration of cellular effect models from computational biological systems into whole-body PBPK models were discussed along with how a thorough mechanistic understanding of physiological processes at different levels of biological organization is necessary to simulate and predict reliably pharmacological and toxicological effects of xenobiotics in living organisms.

Kenichi Umehara (Roche, Switzerland) discussed population and PBPK models for drug-drug interaction trials and trial waivers, and how it is increasingly being utilized to answer clinically untested scenarios. He discussed how the methodology can drive important benefit-risk decisions for drugs across a range of scenarios and how regulating authorities have been supportive of this approach. Dr Umehara presented a series of case examples where PBPK modeling had been employed successfully in drug-drug interaction scenarios. As a future perspective, potential applications of PBPK modeling in drug development were also presented, in alignment with the guideline documents by US Food and Drug Administration (FDA) and European Medical Association (EMA) (European Medicines Agency Science Medicines Health, 2018a).

The FDA describes a PBPK analysis as models and simulations that combine physiology, population, and drug characteristics to mechanistically describe the pharmacokinetic and/or pharmacodynamic behaviors of a drug.

Maxime Le Merdy (Simulations Plus, Switzerland), reviewed how current model applications can be used to support internal development and regulatory decisions. He noted how, throughout a drug's life cycle, predictions derived through PBPK models can be used to support decisions on whether, when and how to conduct certain clinical pharmacology studies, and to support dosing recommendations in product labeling. Dr Le Merdy also presented the final session of the workshop, describing how to obtain biowaivers for clinical trials using PBPK models. He recounted an example of partnership with a pharmaceutical company to construct a model using *in vivo* data collected for a tablet formulation manufactured with a non-particle-engineered drug. Parameter sensitivity analysis and virtual bioequivalence trial simulations were used to demonstrate how a change in manufacturing process would not affect the pharmacokinetic profile.

The workshop closed with an enthusiastic discussion between speakers and participants on key learnings the session had provided, and the general point that PBPK models are only as powerful as the data they are based on. These data were mostly derived from previous clinical trial data, so *in silico* models would not necessarily reduce the number of trials taking place, particularly in the case of rare diseases.

#### Workshop 2: Early Clinical Development of Biologics—What Is So Different About It?

Philip Barrington (Faculty of Pharmaceutical Medicine, UK) discussed the development of biological agents, focusing mainly on monoclonal antibodies and the new therapeutic concepts they introduce. The presentation began with a comprehensive description of biologicals including the potential benefits they bring to the treatment of disease, the challenges often associated with them and their routes of administration. A detailed description was also provided on the structure of different antibody types. A fascinating insight was provided into the development of various agents over the last 5 years and their differentiating characteristics, including checkpoint agonists and antagonists, immune mobilizing monoclonal T-cell bispecific agents, and artificial messenger RNA therapies. Small interfering RNA molecules were also introduced as the new kids on the block.

Dr Barrington described two new developments in the field of biologicals: mucosal injectors to overcome common issues associated with parenteral administration and nanobodies, single domain antibody fragments. The session incorporated a lively discussion over the ethics of exposure of healthy subjects to biological agents during early clinical studies—with their potential to induce long-term changes to subjects that are otherwise healthy, as well as their long-term potential to cause conditions such as cancer.

Stephan Glund (Boehringer Ingelheim, Germany) discussed how biological agents differ markedly in size and complexity from traditional small molecules, noting that biologicals are not simply large chemicals but embody additional levels of complexity. For example, biologicals are manufactured in living cells and are, therefore, subject to a host of post-translational modifications, resulting in product heterogeneity that is impossible to control completely. Each variation has the potential to affect the pharmacokinetic and pharmacodynamic profile of these molecules. This presentation focused mainly on IgG antibodies and Fab-fragments.

It was pointed out by Dr Glund that although these molecules may share similar architecture they can differ significantly in their characteristics. For example, half lives can differ from 24 h to 24 days. Bioanalysis usually involves either liquid chromatography coupled with mass spectrometry or an immunosorbent assay, but the utility of these techniques can be affected by a variety of confounding factors including complex tissue matrices, anti-drug antibodies, and the comparability between different agents.

Biologicals are administered by various different parenteral routes, giving high bioavailability. Tissue clearance is most often *via* the lymph and elimination by cellular uptake followed by proteolytic degradation. Issues remain over the necessity to characterize certain traditional aspects of clinical pharmacology when studying biological agents. This includes the necessity and/or extent of investigation in healthy volunteers, the long-term impact in healthy subjects, and just how long subjects should be followed up after a study ends. Less concern is placed on the need for thorough QT and renal or hepatic studies.

Introducing the current understanding of anti-drug antibodies and immunogenicity, Ann Gils (Therapeutic Drug Monitoring, Belgium) discussed how chronic inflammatory diseases such as rheumatic diseases, spondyloarthritis, inflammatory bowel diseases, and psoriasis have a high prevalence in society and typically start early in life. Consequently, they place a heavy burden on the quality of life and productivity of otherwise relatively young and active individuals. Being selected to interact with highly specific targets, biologicals are generally considered as having low potential for toxicity, particularly with regards to their impact on the liver and kidney.

However, experience has shown that biologicals have the potential to induce immune responses that are moderated by anti-drug antibodies. Immunogenicity is dependent on intrinsic factors such as protein structure, glycosylation, and the covalent attachment of polyethylene glycol to the therapeutic protein and by extrinsic factors such as a patient's genetic make-up and co-medications as well as the route, dose, and frequency of administration. The concentration of the drug and its effectiveness may also be impacted upon by anti-drug antibodies. The formation of anti-drug antibodies appears to be influenced by a variety of factors including co-medication. Dr Gill summarized the types of assays currently available to determine the biologicals clinically and their utility when combined with some unique sampling techniques—such as measuring the presence of biologicals in a fingerprint. The presentation was closed with a summary of modulators of anti-drug antibody production and their clinical potential.

Meagan O'Brien (Regeneron Pharmaceuticals, US) reviewed the optimal approach with regards to pharmacodynamic activity and safety of biologicals in clinical development, the specific nature of biological challenges, and building a knowledge base on how to establish their clinical profile prior to first-in-human studies (FIH). Preclinical investigations require the use of genetically validated targets. However, it is still possible to perform experiments that confirm mechanisms of action, predict safety, and inform the design of future clinical studies. Dr O'Brien gave a summary of the background behind the introduction of the new EMA guidelines of FIH clinical studies and decisions on conduct, dose selection, and permissible levels of exposure (Committee for Medicinal Products for Human Use, 2019). A series of case studies were outlined where biological agents had been investigated and the different challenges experienced and how they were addressed.

Before closing Workshop 2, the attendees participated in an open forum discussion over what had been learned. It was generally agreed that key learnings that have emerged from the increasing number of trials conducted with biological agents in humans are providing the insights required to plan and conduct clinical studies more safely in the future.

### Day 1

EUFEMED president, Hildegard Sourgens (Germany), welcomed attendees, opened the full meeting and explained how the changing landscape of early drug development was the focus of the conference, starting the day with innovations, particularly *in silico* experimentation.

#### Keynote: The Potential Role of Virtual Trials in Early Medicines Development: Beyond Pharmacology to Mechanisms

Adriano Henney (Avicenna Alliance for Predictive Medicine, UK) focused on the use of computational modeling and simulation to interpret quantitative biological information with emphasis on how models can help to understand and inform the drug development process. There has been a growing belief that integrative systems approaches involving computational modeling and simulation can help to unravel these complex biological systems, although adoption and reduction to practice of these approaches has been slow. Dr Henney discussed how ‘virtual trials,’ that run in computer models of human physiological systems, have been successful in the development and testing of medical devices, but application within the pharma sector has been slower. Recently the emergence of Model Informed Drug Discovery and Development (MID3) has demonstrated that computational models are useful in refining classical pharmacometric studies, and also help understand toxicological mechanisms. While this is encouraging, the need remains for *in silico* technologies to help improve our understanding of the pathogenetic mechanisms underpinning complex diseases.

#### Session 1: Current and Future Options for Virtual Trials in Early Medicines Development

*In silico* clinical trials are emerging as a powerful addition to the drug development toolbox. François-Henri Boissel (Novadiscovery, France) provided understanding behind the potential of mechanistic models and their limitations, as well as providing insight into adapting organizations and building the necessary expertise. The systematic use of mechanistic models to explore a large continuum of therapeutic hypotheses prior to human testing has long been expected to de-risk early-stage clinical development, and ultimately reducing the time it takes for medicines to get to patients. Understanding how to use such models and what to expect from them is critical to their widespread adoption. Trust remains low but regulatory agencies are currently working on establishing a framework to include modeling and simulation in their workflows. To be successful in this dawning age of digital evidence, the pharmaceutical industry will need to adapt to this new paradigm by establishing functional teams with the necessary skill sets to exploit the technology as well as develop methods for their verification and validation.

Stig Omholt (Norwegian University of Science and Technology, Norway) noted that there are a lot of buzzwords that surround *in silico* models and artificial intelligence in the business of drug development. However, even though model-based drug development is gaining acceptance as a vital approach in understanding patient risk/benefit and attrition, there is an absence of epistemological thinking. A key characteristic of current drug targeting research is that one employs high-throughput screening of quite simple assays to identify a key molecule involved in a particular metabolic or signaling pathway specific to a disease condition or pathology that may be used to cure patients by inhibiting or enhancing particular biological pathways or processes. This paradigm has also been one of the major drivers behind the development of genomics and the pursuit of large genome-wide association studies, since it was thought that such studies would provide a plethora of putative drug targets for complex diseases. However, it was noted that the advances so far have been modest considering the practical outcomes relative to the enormous resources that have been invested.

Professor Omholt discussed how advanced computational physiology representations embedded in a control-theory framework of what causes and maintains complex disease states may provide the basis of such a new drug targeting paradigm. However, he underlined the complex nature of disease and the necessity of sufficient understanding of the underlying pathology before you can invert the current ‘bottom-up’ approach to discovery, starting from the disease phenotype, working down in a causally cohesive way to the molecular realm where we can link with drug design. Although *in silico* solutions are not yet a panacea for the many challenges faced during early drug development, they represent promising tools with the potential to shorten development timelines and reduce the resource burden and minimize the degree of attrition in drug development.

The session ended with a lively open forum discussion. Key points raised focused on who owns the models that are often created through open access collaborations and how might these systems undergo calibration and/or validation. Concern was also expressed on the availability of team members capable of programming, validating, and interrogating the *in silico* models. It was generally agreed that model development is an iterative process where new ‘aspects’ or ‘influences’ need to be added, and with the underlying paradigm changing over time providing further insights become available on the disease/condition and we gain more information on the characteristics of the disease. The situation was left with an open question, what will the FDA or EMA say if models become so powerful that they start to markedly reduce the number of subjects taking part in clinical studies (and therefore, the overall size of safety databases)?

#### Session 2: Trends and Innovation

At the beginning of the innovation session, Andrew Warrington (Switzerland) representing #WeAreNotWaiting, raised the possibility that informed and motivated patients will, in the future, start to engage in and disrupt their own treatment pathways through the equipment often used to manage their disease. Mr Warrington detailed his own (and his groups) dissatisfaction with type-1 diabetes treatment technology, namely artificial pancreas devices. When the technology to make such a device became widely available and affordable, a loose affiliation of patients took it upon themselves to build their own devices that better mimicked the physiological management of insulin, going on to prove that their own algorithms were safe and effective. They also ‘hacked’ commercial technologies to modify devices so that they were better adjusted to disease requirements. Mr Warrington demonstrated how group-thinking by those outside the pharma/device industry are influencing progress—demanding better therapies and taking the initiative where necessary.

Roman Galetto (Cellectis SA, France) discussed engineering allogeneic immune cells to generate off-the-shelf CAR T-cell immunotherapies. Chimeric antigen receptors (CARs) are engineered molecules that, when present at the surface of T-cells, enable them to recognize specific proteins or antigens that are present on the surface of target cells. These receptors provide T-cells with a specific targeting mechanism to seek out and destroy the tumor cells bearing a selected antigen associated with that tumor. CART immunotherapies are one of the most promising approaches to fighting cancer, and have shown long-term durable remission and remarkable response rates in patients with refractory leukemia. Most CAR T-cell therapy products are currently generated from autologous cells, with the limitation that they have to be manufactured on a ‘per patient’ basis, which remains expensive and difficult to implement for lymphogenic and critically ill patients.

Dr Galetto discussed a standardized platform for manufacturing CAR T-cells from healthy donors to generate allogeneic ‘off-the-shelf’ products while inactivating aspects of the T-cell believed to be responsible for mediating graft versus host diseases. The possibility was raised that universal CAR T-cells can be industrialized and thereby standardized with consistent pharmaceutical release criteria, over time and from batch to batch. It was agreed that this would represent a paradigm shift in terms of ease of use, availability and the drug pricing challenges associated with CAR-T therapies.

#### Session 3: Posters and Oral Presentations

Researchers were given the opportunity to submit abstracts to be displayed as posters. After evaluation by the scientific committee, five projects were presented as oral presentations and the remainder as posters. All abstracts are published in https://www.eufemed.eu/wp-content/uploads/EUFEMED-Final-Programme-and-Abstract-Book.pdf.

#### Parallel Breakout Sessions on Current Challenges in Early Phase Development

The final session of Day 1 involved four parallel breakout sessions, each dealing with specific issues currently challenging early phase development.

The first breakout session hosted by Robert Rissmann (Leiden University Medical Centre, The Netherlands) and Ingrid Klingmann (PHARMAPLEX bvba, Belgium) considered the potential impact of digital support on study performance in early phase development, extending from recruitment to remote data collection. The current breakthrough of mobile technologies in daily life represents a major opportunity in terms of development of clinical trials. They could reduce the requirement for clinic visits during trials, gradually changing to the patients' home setting with the collection of more ‘real world’ information. Almost all parameters can be monitored comprehensively including vital signs, movement patterns, social behavior, activity, specific symptoms such as tremor, as well as treatment adherence, symptom diaries (etc). In order to take full advantage of the potential offered by mobile technology it will be necessary to develop robust tools and applications.

The EU Clinical Trials Regulation 536/2014 mandates a summary of clinical trials results that is understandable for laypersons (European Medicines Agency Science Medicines Health, 2018b). Lay summaries are intended to increase research transparency and to provide the public with the key information about the trial. Kerstin Breithaupt-Grögler, Germany, and Leonie Leithold (Boehringer Ingelheim Pharma GmbH & Co. KG, Germany) reviewed possible consequences of the regulation for phase I trials. They also discussed the 10 elements that must be covered in a lay summary listed in Annex V of the regulation. Lay summaries address the general public as well as participating subjects and patients. A summary must be prepared for every clinical trial and be posted on the EU Portal within 12 months after the end of the study. For phase I trials without therapeutic intent, this timeline may be extended up to 30 months. Shorter timelines apply for pediatric trials (6 months).

Available guidance documents regarding structure and content as well as literacy and numeracy principles applicable for a lay summary were addressed. An example summary of a typical pharmacokinetic trial in healthy subjects was presented. Questions from the audience concerned the description of primary and secondary endpoints, the sociocultural aspects of data presentation, wording of safety aspects and formatting. When the lay summary development process should start was discussed as well as who should write the first draft.

Transparency is currently a major topic of debate in clinical research. Companies are being required to register clinical drug trials in public registries. Gerhard Koëter (Netherlands) and Sander van den Bogert (Apotheek Boekel, Netherlands) reviewed the current transparency requirements for phase I trials. The session covered recent research into how phase I trials are performing in terms of transparency *versus* other trial types, and why phase I trials in oncology are doing better. A brief overview was provided of the current legal requirements and of expected future changes in transparency policies with the implementation of changes in EU regulations and opening of the EU portal (European Medicines Agency Science Medicines Health, 2018b; European Medicines Agency Science Medicines Health, 2019). Consideration was given to whether too much transparency might harm the business model of the industry and our understanding of what defines commercially confidential information. Concerns were expressed over suggestions that industry should be required to publish clinical trial protocols and investigator's brochures as well as the informative role of social media over academic publications. Overall, the session invited the audience to participate in an interactive discussion about these and other issues relating to transparency.

Sylvie Rottey (Drug Research Unit Ghent, Belgium) and Jan de Hoon (University of Leuven, Belgium) provided a fascinating perspective of what we should consider to be acceptable or ethical to test in healthy subjects. The session opened by considering past failures in FIH trials and what we might learn from incidents like the BIAL trial (Bonini and Rasi, 2016). The presenters defended the safety record of early phase experiments and illustrated their position with data from a survey of over 21,000 participants (between 2009 and 2015). As a result, 0.38% of the healthy volunteers suffered from a study related serious adverse event.

It was proposed that clinical trials remain the best tool for gathering the evidence needed for drug approval and that they remain an appropriate clinical practice, though consideration was given to the challenge of including healthy volunteers in phase I trials of certain toxic therapies such as anti-cancer treatments. It was reiterated that the critical aspect is in managing the balance between the knowledge gained and the risk experienced. It was noted that *primum non nocere* remains the underlying principal and the uncrossable threshold of everyone involved in medicine.

Can we test potential new cancer drugs in healthy volunteers? How acceptable is taking a biopsy, or a lumbar puncture, implanting a device, etc.? Depending on preclinical investigations, the nature, and mechanism of action of the medicinal product, one should build a clear rationale why healthy volunteers should undergo specific tests or investigations. If a clear rationale is present in the development of a new drug, these investigations should be defended in the protocol, and as such explained during the informed consent procedure.

### Day 2

#### Session 4: Update on Regulatory Consideration for Early Clinical Development (Including Brexit)

Ian Rees (MHRA, UK) provided a summary of the MHRA's current perspective regarding the regulatory landscape in the UK following Brexit. As it stands currently, the UK will continue with full participation in the EU regulatory network. Mr Rees noted that both the EMA and MHRA planned for a continued alignment. The MHRA expects that an agreement will be achieved—though they have produced contingency publications that will provide guidance in the event of a ‘no deal’ Brexit. Clinical sites in the UK, and UK data, will still be valid for multinational trials. Modifications will be expected in the area of marketing authorizations and investigational medicinal products (packaging, the qualified person [QP] function and movement across boarders). The MHRA is focused on maintaining the UK supply chain, and it is expected that the UK will continue to be part of the Pharmaceutical Inspection Convention and Pharmaceutical Inspection Co-operation Scheme as well as the International Coalition of Medicines Regulatory Authorities work sharing initiative. Mr Rees also gave a summary of the MHRA's corporate plan for the 2018–23 period, underlying its ambition to support and enhance innovation and accelerate routes to market as well as to benefit public health and serve as a magnet to attract the life sciences industry to work in the UK.

Fergus Sweeney (European Medicines Agency, Netherlands) gave an update on the EU's stance on clinical trial regulation, departure of the UK from the EU, the EU Network Training Centre, and renovation of ICH and Good Clinical Practice (GCP). He also gave a view on the future regulatory science strategy for the EMA. Focus was provided on the Clinical Trials Information System (CTIS), intended to serve as a regulatory resource for EU supervisory authorities and clinical trial sponsors. The CTIS will become the single entry point for submitting clinical trial information and as such represents a real opportunity for the EU to innovate and lead the way in clinical study oversight. Mr Sweeney gave an overview of the activities the site will manage, the infrastructure involved and the current stage of development.

In discussing Brexit, Dr Sweeney reiterated the position of the MHRA while calling for all pharmaceutical companies in the EU to continue with their preparations for the UK's withdrawal, at which point the UK will adopt a third country position (European Medicines Agency Science Medicines Health, 2018c; Medicines and Healthcare products Regulatory Agency, 2019). He noted that science and research are international, and any trial data will be accepted in EU marketing authorization applications so long as they meet scientific and ethical standards equivalent to those that apply within the EU. The EU is also working on the renovation of ICH GCP, to prepare the regulatory landscape for future medicines, future trial designs, and future data sources. The emphasis is on the role of achieving quality through good design. Revisions in the International Conference on Harmonisation of Technical Requirements for Registration of Pharmaceuticals for Human Use—General Considerations for Clinical Trials (E8), and Guideline for Good Clinical Practice (E6) are expected to proceed from 2019 to 2026, respectively.

The aim is to support innovative approaches to clinical trials including: facilitating a broad range of study designs and data sources; upfront assessment of risks specific to a study design, protocol and procedures, proportionate risk management and controls focusing on critical study elements, and use of technological tools to ensure robust conduct, oversight, and reporting.

The EMA feels that the greatest challenge will be in change management—adjusting behaviors and attitudes. However, it predicts that those who embrace new approaches and seek to make them work will benefit the most. In concluding his presentation, Dr Sweeney noted that the EMA foresees itself adopting an evolving role for medicines regulatory agencies—serving as both a gatekeeper (evaluation and supervision) and an enabler (supporting research and innovations and connecting stakeholders).

Nick Sykes (Pfizer, UK) gave an update on regulatory considerations for early clinical development, including Brexit, from the viewpoint of the larger players in the pharmaceutical industry. In considering the impact of pre-Brexit regulatory preparations, the main focal points have involved labeling/packaging, QP release, and legal representation. He recounted how, for Pfizer, this had involved approximately 650 submissions relating to 107 protocols across 26 EU/European Economic Area markets.

Mr Sykes described how the industry currently felt there was limited cause for concern as they had received pragmatic guidance from the MHRA, their clinical trial market and regulation was national and UK trials are being conducted to existing international standards (and thus UK data will remain relevant post-Brexit). The key issues, he felt, lay around potential border delays and the need for duplication of activities between the UK and Europe (e.g., QP certification, safety reporting.)

On reviewing the impact of the EU First in Human Guidance (Committee for Medicinal Products for Human Use, 2019), Mr Sykes presented the results of the EUFEMED forum conducted in September 2018 and reported in April 2019 Breithaupt-Grögler et al. (2019). In terms of the future of Clinical Trials Regulation and the adoption and development of different types of trials, Mr Sykes concluded that we are facing a different way of working in the EU but the timeline for change remains unspecified.

#### Session 5: How to Be Prepared

Nuria Kotecki (Jules Bordet Institute, Belgium) focused on new approaches and designs in oncology. Advances in the understanding of tumor biology have changed the landscape of clinical research and resulted in evolving treatment strategies such as precision medicine implying the use of targeted agents and activating the immune system against cancer using immunotherapy. Dr Kotecki discussed how the move from empirical cytotoxic to molecular and immunological therapeutic approaches has impacted clinical trial designs. She provided the examples of adapted study designs and the changing definitions and dose-limiting toxicities and endpoints. However, Dr Kotecki expressed the opinion that these novel approaches remain underused. The presentation emphasized the need to engage more readily with innovative strategies, approaches and study designs in early drug development in oncology.

Current challenges in exploratory clinical research were discussed by Maarten Van den Boer (Janssen Pharmaceutica, Belgium), focusing on how exploratory clinical research is changing. Throughout the industry we are seeing ever more complex trial designs with their execution also becoming more complex with each innovation. There is a high need for early data readouts in the patient population to reduce the overall drug development cost and timelines. Getting patients in early phase I trials turns out to be very challenging. These recruitment problems can be protocol-, patient- and investigator-related. Dr Van de Boer reflected on how underlying recruitment problems might be identified. He also questioned whether we should change the way we work. Alternate approaches of how early phase patient involvement could be managed and executed were discussed.

Virginia Parks (Takeda, USA) provided an industry insight into current perspectives on digital biomarker development in early clinical research. She detailed how wearable technology is being increasingly implemented in clinical drug development—data from clinicaltrials.gov identified 330 clinical trials that included their use in 2017. Technology allows the collection of observable data non-invasively, in real-time, in a patient's natural setting to enhance our understanding of the effects of treatment and how symptoms may change over time. Combined with predictive analytical techniques such as machine learning, wearable technologies promise to contribute to the advancement of innovative therapeutics as effective treatment solutions through novel endpoint, or digital biomarker discovery.

Although there is much enthusiasm for its use in medical research, more rigorously gathered data is needed for the field to progress and to establish valid methodologies. The considerations and challenges for development of a novel digital biomarker were discussed, and how these pertain to clinical pharmacologists and pharmacometricians who are frequently involved in decision-making on the right dose, right patient, study design, and trial progression.

Many insights can be harvested from the use of wearable technologies, as illustrated by recent case study examples e.g., digitally acquired motor symptom data in Parkinson's disease. However, it is noted that most of the data currently being produced is from observational, non-randomized small-scale studies without placebo control. There are therefore real and perceived scientific and operational challenges related to the implementation of a digital biomarker strategy in early clinical development. The obstacles to greater adoption and acceptance are discussed, including current regulatory guidelines, frameworks for industry, and thoughts for future developments in this nascent field of research.

## Conclusion

The 2019 EUFEMED conference was well attended and facilitated valuable cross-discipline interaction. It provided a unique forum for learning, not only providing information on new topics, but also expanding the existing knowledge of all attendees. The conference achieved its objective of focusing on early clinical drug development in a changing scientific and regulatory environment. In closing the meeting, the president elect, Yves Donazzolo (Eurofins Optimed, France), summarized how the topics discussed during the meeting served to foster a shared appreciation of the innovative nature of the early clinical development space, and welcomed the commitment of all parties to addressing concerns over risk and improving our understanding of the challenges ahead.

## Author Contributions

This work is written from an audience perspective. All authors were part of the scientific and/or organizing committee and contributed to the content of the conference. They were all able to interpret the presentations, critically revised the manuscript, gave final approval and agreed to be accountable for all aspects of the work in ensuring that questions related to the accuracy or integrity of any part of the work are appropriately investigated and resolved. DD and TH drafted the manuscript.

## Funding

The conference was made possible by: Abide Therapeutics, Aepodia, Arensia Exploratory Medicine, Biotrial, Celerion, CTC North, Eurofins Optimed, Foundry Health, Lixoft, MSD & Co, Niche Science & Technology, PRA Health Sciences, PhinC Development, Syndesi Therapeutics, Richmond Pharmacology, Sanofi, SGS, Verified Clinical Trials, Welch Allyn. These companies were exhibitors at the conference or provided an unconditional grant.

## Conflict of Interest

Author TH is employed by the company, Niche Science & Technology Ltd.

The remaining authors declare that the research was conducted in the absence of any commercial or financial relationships that could be construed as a potential conflict of interest.
